# Development and Internal Validation of an MRI-Based Diagnostic Prediction Model for Differentiating Cystic Pituitary Adenoma and Rathke’s Cleft Cyst

**DOI:** 10.3390/medicina62040659

**Published:** 2026-03-31

**Authors:** Elif Kapitasi Kerter, Ozlem Unal, Ayse Nur Sirin Ozcan

**Affiliations:** 1 Department of Radiology, Zonguldak Ataturk State Hospital, Zonguldak 67030, Türkiye; 2 Department of Radiology, Ankara Yıldırım Beyazıt University, Ankara 06300, Türkiye; 3 Department of Radiology, Bilkent City Hospital, Ankara 06530, Türkiye

**Keywords:** cystic pituitary adenoma, magnetic resonance imaging, Rathke’s cleft cyst, differential diagnosis

## Abstract

*Background and Objectives*: Differentiation between cystic pituitary adenoma and Rathke’s cleft cyst (RCC) is clinically important because these lesions require different treatment strategies. This study aimed to develop and internally validate an MRI-based multivariable diagnostic prediction model to differentiate cystic pituitary adenoma from Rathke’s cleft cyst before treatment. *Materials and Methods*: A retrospective analysis was performed on 56 adult patients (27 cystic pituitary adenomas and 29 RCCs) who underwent pituitary MRI between 2019 and 2021. MRI examinations were independently evaluated for ten predefined imaging features. Diagnoses were established using histopathology or a validated clinical–radiological diagnostic algorithm. Interobserver agreement and diagnostic performance were analyzed using multivariable logistic regression, with internal validation performed using bootstrap resampling. *Results*: Interobserver agreement was excellent (κ (kappa) = 0.81–1.0). Fluid–fluid level, hypointense rim on T2-weighted images, septation, and paramedian coronal location were significantly associated with cystic pituitary adenoma. In contrast, spontaneous T1-weighted hyperintensity, intracystic nodule, and midline sagittal location were more frequently observed in RCC. The final multivariable model demonstrated excellent discrimination (AUC = 0.91), with stable performance after bootstrap validation (optimism-corrected AUC = 0.88). *Conclusions*: The proposed MRI-based multivariable prediction model demonstrated high discrimination and provides a structured approach for estimating the probability of cystic pituitary adenoma using routinely available MRI features. Such an approach may help reduce unnecessary surgical interventions in patients with Rathke’s cleft cyst while facilitating appropriate treatment planning for cystic pituitary adenomas. However, external validation in larger cohorts is required before routine clinical implementation.

## 1. Introduction and Aim

Pituitary adenoma is the most common intrasellar pathology originating from adenohypophysis. Pituitary adenomas account for approximately 10–15% of all intracranial neoplasms [1]. Pituitary adenomas may contain a partially cystic component or may be purely cystic. Another cystic lesion of the pituitary gland, Rathke’s cleft cyst, is a benign epithelial cyst thought to originate from remnants of Rathke’s pouch [2]. Depending on its content, Rathke’s cleft cyst may demonstrate various signal intensities on both T1- and T2-weighted images [3]. Rathke’s cleft cyst may sometimes appear hyperintense on T1-weighted images and hypointense on T2-weighted images due to high intracystic protein content, thereby mimicking hemorrhagic cystic pituitary adenoma [4]; this makes differentiation between cystic pituitary adenoma and Rathke’s cleft cyst using magnetic resonance imaging challenging.

Cystic pituitary adenoma and Rathke’s cleft cyst are both common intrasellar cystic lesions; however, they have different treatment strategies and prognoses in clinical practice [5,6]. While routine magnetic resonance imaging and clinical follow-up are sufficient for incidentally detected asymptomatic Rathke’s cleft cysts, the relatively rare symptomatic lesions are treated surgically [7,8,9]. There is no indication for surgical intervention in incidentally detected asymptomatic Rathke’s cleft cysts [6]. Differentiation between these two lesions is important for preoperative treatment planning in pituitary adenomas. In cases requiring surgery, partial resection of the cyst wall or drainage of the cyst content is usually sufficient for Rathke’s cleft cysts, whereas complete resection may be required for cystic pituitary adenomas not only to reduce mass effect but also to correct hormonal disturbances [10]. Unnecessary surgical excision of a Rathke’s cleft cyst may lead to serious complications such as cerebrospinal fluid leakage, infection, and hypothalamic injury [7]. Therefore, obtaining an accurate preoperative diagnosis is of great importance for neurosurgeons in order to determine appropriate surgical indications and to plan the optimal surgical procedure.

Although endocrine tests are effective in differentiating cystic pituitary adenoma from Rathke’s cleft cyst, magnetic resonance imaging remains a vital diagnostic tool in the diagnosis of these two lesions, particularly in cases of non-functioning pituitary adenomas [10]. In recent years, magnetic resonance imaging features of cystic pituitary adenoma and Rathke’s cleft cyst have been well documented. For example, the presence of a fluid–fluid level, off-midline location, septation, and a hypointense rim on T2-weighted images are considered highly specific for cystic pituitary adenoma, whereas an intracystic nodule is frequently observed in Rathke’s cleft cysts [11,12].

In many cases, however, these two lesions are difficult to distinguish based on magnetic resonance imaging findings alone [13]. Cystic pituitary adenomas may demonstrate high signal intensity (SI) on T1-weighted images due to subacute hemorrhage or low signal intensity (SI) due to liquefaction, and may also show high SI on T2-weighted images due to liquefaction or low SI due to necrosis secondary to chronic hemorrhage [13]. These radiological findings may mimic Rathke’s cleft cysts with varying protein content [4]. Therefore, overlapping imaging features pose a challenge in the differential diagnosis using magnetic resonance imaging.

One of the most detailed studies conducted to date on the differentiation between cystic pituitary adenoma and Rathke’s cleft cyst is the study by Park et al. published in 2015 [11]. In their pathology-correlated study, they proposed a diagnostic algorithm for 54 cystic pituitary adenomas and 28 Rathke’s cleft cysts. In this algorithm, the diagnostic value of magnetic resonance imaging findings such as fluid–fluid level, hypointense rim on T2-weighted images, septation, paramedian location, presence or absence of an intracystic nodule, suprasellar extension, size change, and signal change was evaluated [11]. Park et al. compared their algorithm with pathological results and were able to correctly classify 79 of 82 cases (96.3%) in their study population [11].

Spontaneous hyperintensity on T1-weighted images, maximum lesion diameter values, and sagittal location characteristics were also included in the present study, as these features may contribute to the differential diagnosis.

In neuroradiological practice, spontaneous T1-weighted hyperintensity is more frequently observed in Rathke’s cleft cysts than in cystic pituitary adenomas [4]. Although cases of cystic pituitary adenomas demonstrating T1-weighted hyperintensity have been described in the literature [13], there are no studies evaluating the diagnostic value of this imaging feature in differentiating between these two lesions.

In neuroradiological practice, Rathke’s cleft cysts are generally located in the midline on sagittal images [4], whereas cystic pituitary adenomas show variable locations [10]. Although the literature reports that the location of Rathke’s cleft cysts may vary, midline location on sagittal images was hypothesized to be potentially useful for differentiating Rathke’s cleft cyst from cystic pituitary adenoma. In addition, although only a limited number of studies exist in the literature, paramedian location of cystic pituitary adenomas on coronal images has been frequently reported in neuroradiological practice [10], and this feature was considered potentially valuable in the differential diagnosis.

Unlike previous studies that evaluated individual MRI features descriptively, the present study aims to develop and internally validate a multivariable MRI-based diagnostic prediction model integrating multiple imaging characteristics into a unified predictive framework. By combining several MRI parameters within a logistic regression model and assessing its discrimination, calibration, and clinical utility, this study provides a more systematic and clinically applicable approach for differentiating cystic pituitary adenoma from Rathke’s cleft cyst in routine neuroradiological practice. Such multivariable approaches may improve the reproducibility of radiological decision-making compared with reliance on individual imaging signs alone.

The aim of this study was to determine which magnetic resonance imaging features are most useful in differentiating cystic pituitary adenoma from Rathke’s cleft cyst and to develop an internally validated multivariable MRI-based diagnostic prediction model for preoperative differentiation of these lesions.

## 2. Materials and Methods

### 2.1. Study Design

The study was initiated after obtaining approval from the Ankara Bilkent City Hospital Scientific Research Ethics Committee (TABED). This retrospective study was conducted in accordance with the principles of the Declaration of Helsinki and the Good Clinical Practice guidelines. The study followed the TRIPOD reporting guideline for prediction model studies. This research received no external funding.

### 2.2. Patient Selection

Patients who underwent pituitary magnetic resonance imaging for any indication at the Department of Radiology, Ankara City Hospital, between 2019 and August 2021 and who were aged 18 years or older were included in the study. Patients were identified through a retrospective review of pituitary MRI examinations in the Picture Archiving and Communication System (PACS). Images were initially evaluated by an experienced radiologist, and cases with cystic lesions detected in the pituitary gland were included.

Lesions containing solid components or contrast-enhancing solid components, patients who had undergone surgery without available preoperative imaging, and images with artifacts that impaired adequate evaluation were excluded.

### 2.3. MRI Protocol

All MRI examinations were performed using a 3.0-T General Electric Signa Pioneer scanner (GE Healthcare, Milwaukee, WI, USA) equipped with a dedicated head coil at Ankara City Hospital. A standardized pituitary MRI protocol was applied to all patients.

The imaging protocol included pre-contrast T1-weighted sagittal and coronal sequences, pre-contrast T2-weighted coronal sequences, post-contrast T1-weighted sagittal and coronal sequences, and dynamic contrast-enhanced coronal T1-weighted sequences.

For pre-contrast sagittal T1-weighted fast spin-echo (FSE) sequences, the slice thickness was 2.5 mm with a slice gap of 0.5 mm, repetition time (TR) was 556 ms, echo time (TE) was 16.45 ms, and the field of view (FOV) was 150 mm.

For pre-contrast coronal T2-weighted fast spin-echo (FSE) sequences, the slice thickness was 2 mm with a slice gap of 0.5 mm, TR was 2522 ms, TE was 110 ms, and the FOV was 150 mm.

For pre-contrast coronal T1-weighted fast spin-echo (FSE) sequences, the slice thickness was 2 mm with a slice gap of 0.5 mm, TR was 532 ms, TE was 10.75 ms, and the FOV was 150 mm.

For post-contrast sagittal T1-weighted fast spin-echo (FSE) sequences, the slice thickness was 2.5 mm with a slice gap of 0.5 mm, TR was 556 ms, TE was 16.45 ms, and the FOV was 150 mm.

For dynamic contrast-enhanced coronal T1-weighted sequences, the slice thickness was 2 mm with a slice gap of 0.5 mm, TR was 550 ms, TE was 8.83 ms, and the FOV was 160 mm. A total of 11 consecutive dynamic series were obtained.

A gadolinium-based contrast agent was administered intravenously at a dose of 0.1 mmol/kg (0.2 mL/kg body weight) at an injection rate of 2 mL/s, with a maximum dose of 20 mL.

### 2.4. Image Evaluation

All conventional MRI images were initially evaluated by an experienced radiologist, and patients with cystic lesions in the pituitary gland without contrast-enhancing solid components were included in the study. Images containing contrast-enhancing solid components or artifacts that impaired evaluation were excluded.

Among the included patients, histopathological confirmation was available in 13 cases. For the remaining patients who did not undergo surgery, diagnosis was based on clinical findings, endocrine evaluation, and longitudinal imaging follow-up using the diagnostic algorithm proposed by Park et al. [11]. This approach reflects routine clinical management of asymptomatic cystic pituitary lesions, where surgical confirmation is not always available.

For patients without histopathological confirmation, lesion classification was based on clinical findings, endocrine laboratory results, and longitudinal imaging follow-up. The median follow-up duration for these patients was 12 months (range: 6–24 months). Stability or typical evolution of imaging findings during follow-up was considered supportive of the final classification.

For these patients, consensus classification as Rathke’s cleft cyst or cystic pituitary adenoma was performed by the investigators based on clinical–laboratory data and follow-up imaging findings. The diagnostic algorithm developed by Park et al. [11] was used for consensus evaluation.

We acknowledge that the use of imaging follow-up and an imaging-based diagnostic algorithm for case classification may introduce a potential risk of diagnostic misclassification and circular reasoning, since some of the evaluated imaging features overlap with those used for diagnostic consensus. To mitigate this risk, the radiological feature assessment for the prediction model was performed independently and in a blinded manner, in a separate session from the consensus classification process.

In a separate session from the consensus evaluation, the radiological features of these lesions were independently evaluated by two investigators (the first investigator was an experienced radiologist, and the second investigator was a fifth-year radiology resident) using all MRI sequences.

Before the independent image evaluation, both investigators reviewed the predefined imaging criteria used in the study to ensure consistent interpretation of MRI features. During the evaluation process, the observers were blinded to all clinical information, endocrine laboratory findings, follow-up data, and the final diagnostic classification. In addition, the observers were unaware of each other’s interpretations and performed all assessments independently.

The following features were recorded for each lesion: presence or absence of fluid–fluid level; spontaneous hyperintensity on T1-weighted images; hypointense rim on T2-weighted images; septation; paramedian location on coronal images; midline location at the pars intermedia on sagittal images; presence of an intracystic nodule; size change during follow-up; signal change during follow-up; and maximum lesion diameter.

Maximum lesion diameter was defined as the largest measurement obtained from the transverse and superoinferior diameters on coronal images and the anteroposterior diameter on sagittal images.

Size change during follow-up was defined as a measurable increase or decrease in the maximum lesion diameter compared with the baseline MRI examination. Signal change was defined as a qualitative alteration in the signal intensity characteristics of the cystic component on follow-up MRI sequences relative to the initial examination. These changes were evaluated by comparing the baseline MRI with subsequent follow-up examinations.

### 2.5. Model Development

A multivariable logistic regression approach was used to develop the diagnostic prediction model. Candidate predictors were identified based on previously reported MRI features and their clinical relevance in differentiating cystic pituitary adenoma from Rathke’s cleft cyst.

Variables demonstrating statistical significance in univariate analysis, together with clinically meaningful imaging features, were considered for inclusion in the multivariable model. To minimize the risk of model overfitting, the number of predictors included in the final model was deliberately restricted according to the events-per-variable principle. Given the number of events in the dataset, the final model was limited to a small set of key imaging predictors in order to maintain model stability and reduce the likelihood of regression coefficient instability.

Binary logistic regression analysis was performed with cystic pituitary adenoma as the dependent variable. Odds ratios (ORs) with 95% confidence intervals (CIs) were calculated. All predictors were entered into the model as binary variables.

Although some predictors did not remain statistically significant in the final multivariable model, they were retained because of their previously reported diagnostic relevance in the neuroradiological literature and their potential contribution to the clinical interpretability of the prediction model. Retaining these variables allowed the model to incorporate imaging features that may provide complementary diagnostic information when considered together with other predictors.

Model discrimination was evaluated using receiver operating characteristic (ROC) curve analysis and the area under the curve (AUC).

Internal validation of the prediction model was performed using bootstrap resampling with 1000 iterations. In each bootstrap sample, the prediction model was refitted and its performance was evaluated both in the bootstrap sample and in the original dataset. The average difference between these performances was used to estimate model optimism. The optimism-corrected AUC was obtained by subtracting the estimated optimism from the apparent AUC of the original model.

Model calibration was assessed using the Hosmer–Lemeshow goodness-of-fit test and calibration plots. Clinical utility of the prediction model was evaluated using decision curve analysis.

A nomogram based on the final multivariable logistic regression model was developed to provide a simplified graphical tool for estimating the probability of cystic pituitary adenoma in individual patients. The nomogram was constructed using the regression coefficients of the final model predictors.

Model development, internal validation, and reporting were conducted in accordance with the TRIPOD recommendations for prediction model studies.

### 2.6. Statistical Analysis

Before statistical analysis, all variables were reviewed for completeness. No substantial missing data were identified among the imaging variables included in the analysis; therefore, a complete-case analysis was performed without data imputation.

Descriptive statistics were used to summarize demographic and imaging characteristics. Continuous variables were expressed as mean ± standard deviation or median (range), as appropriate, whereas categorical variables were presented as frequencies and percentages.

Group comparisons between cystic pituitary adenoma and Rathke’s cleft cyst were performed using the chi-square test or Fisher’s exact test for categorical variables and the Mann–Whitney U test for continuous variables.

Model discrimination was assessed using receiver operating characteristic (ROC) curve analysis and the area under the curve (AUC). Internal validation was performed using bootstrap resampling with 1000 iterations.

All statistical tests were two-sided, and a *p* value < 0.05 was considered statistically significant. Statistical analyses were performed using IBM SPSS Statistics for Windows, version 26.0 (IBM Corp., Armonk, NY, USA).

## 3. Results

A total of 56 patients were included in the study, of whom 27 (48.21%) were classified as having cystic pituitary adenoma and 29 (51.79%) as having Rathke’s cleft cyst. In the cystic pituitary adenoma group, 8 patients (29.63%) were male and 19 (70.37%) were female, whereas the Rathke’s cleft cyst group included 5 male (17.24%) and 24 female patients (82.76%). The difference between groups was not statistically significant (*p* = 0.273).

The mean age was 39.7 ± 15.11 years (range: 18–65) in the cystic pituitary adenoma group and 32.79 ± 9.86 years (range: 19–54) in the Rathke’s cleft cyst group, with no statistically significant difference between groups (*p* = 0.144). Demographic characteristics are summarized in Table 1.

### 3.1. Interobserver Agreement

Interobserver agreement between the two readers was excellent, with kappa values ranging from 0.81 to 1.00 (*p* < 0.05 for all comparisons). The highest agreement was observed for the identification of a hypointense rim on T2-weighted images (κ = 1.00). Detailed interobserver agreement results are presented in Table 2.

### 3.2. Comparison of MRI Features Between Groups

**Figure 1:**
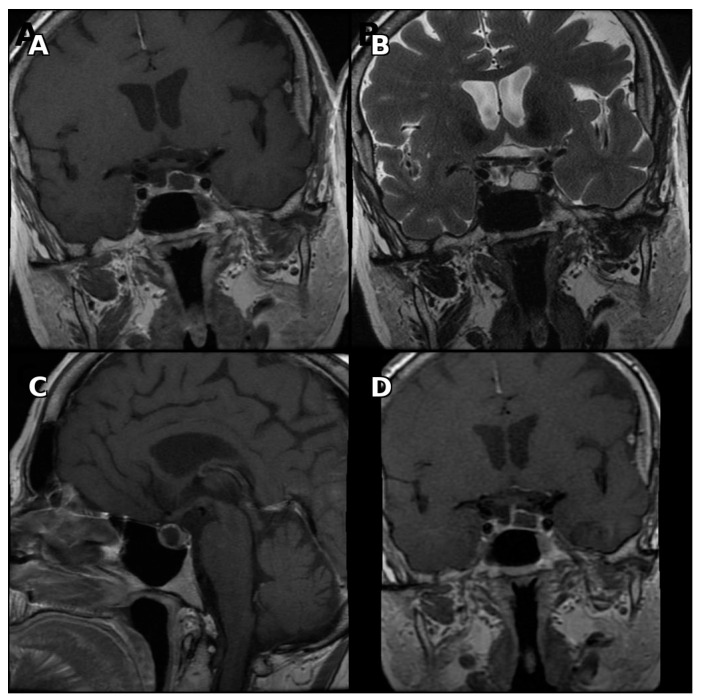
Pituitary MRI of a patient with cystic pituitary adenoma. (**A**) Contrast-enhanced coronal T1-weighted image demonstrating paramedian localization. (**B**) Coronal T2-weighted image demonstrating a hypointense rim surrounding the cystic lesion. (**C**) Contrast-enhanced sagittal T1-weighted image demonstrating anterior localization. (**D**) Delayed contrast-enhanced coronal T1-weighted image demonstrating lack of internal enhancement.

**Figure 2:**
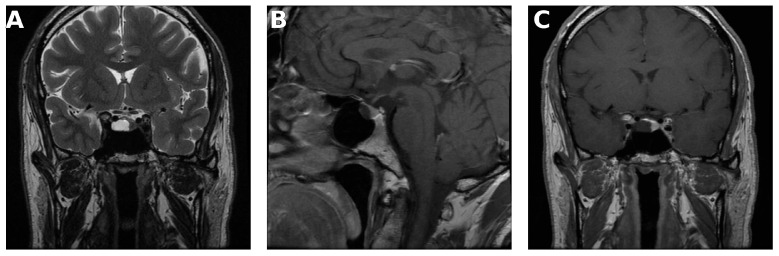
(**A**) Coronal T2-weighted image demonstrating thin internal septations within a cystic pituitary adenoma. (**B**) Contrast-enhanced sagittal T1-weighted image demonstrating anterior localization of the lesion. (**C**) Contrast-enhanced coronal T1-weighted image demonstrating paramedian localization.

**Figure 3:**
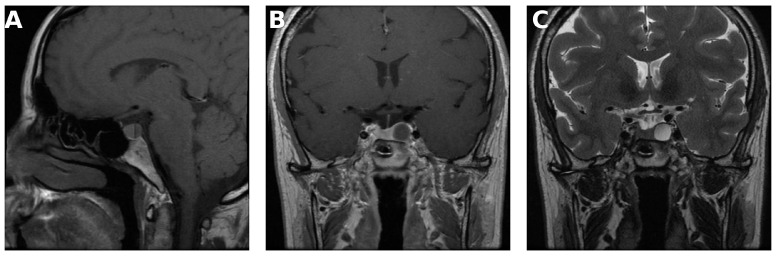
(**A**) Sagittal T1-weighted image demonstrating a fluid–fluid level within a cystic pituitary adenoma. (**B**) Contrast-enhanced coronal T1-weighted image demonstrating paramedian localization. (**C**) Coronal T2-weighted image demonstrating a hypointense rim.

Fluid–fluid level, hypointense rim on T2-weighted images, and septation were observed significantly more frequently in cystic pituitary adenomas (Figure 1, Figure 2 and Figure 3). Representative examples are shown below.

**Figure 4:**
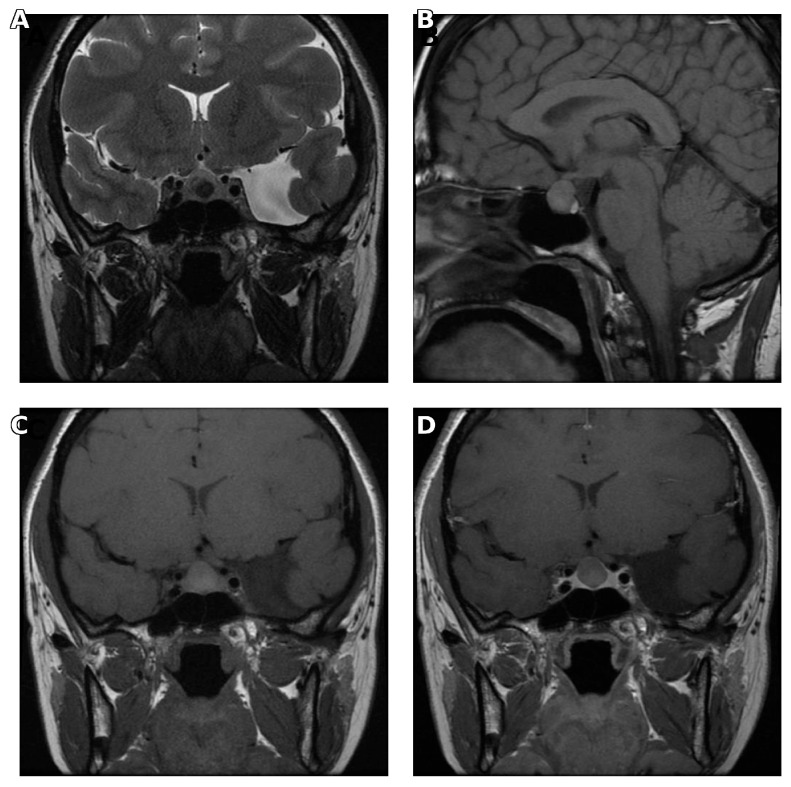
(**A**) Coronal T2-weighted image demonstrating an intracystic hypointense nodule. (**B**) Contrast-enhanced sagittal T1-weighted image demonstrating a midline-located Rathke’s cleft cyst. (**C**) Coronal T1-weighted image demonstrating the intracystic nodule. (**D**) Contrast-enhanced coronal T1-weighted image demonstrating no enhancement of the intracystic nodule.

Intracystic nodules were more commonly identified in Rathke’s cleft cysts (*p* = 0.023, *p* = 0.018, *p* < 0.001, and *p* < 0.001, respectively) (Table 3). Representative example is shown in Figure 4.

**Figure 5:**
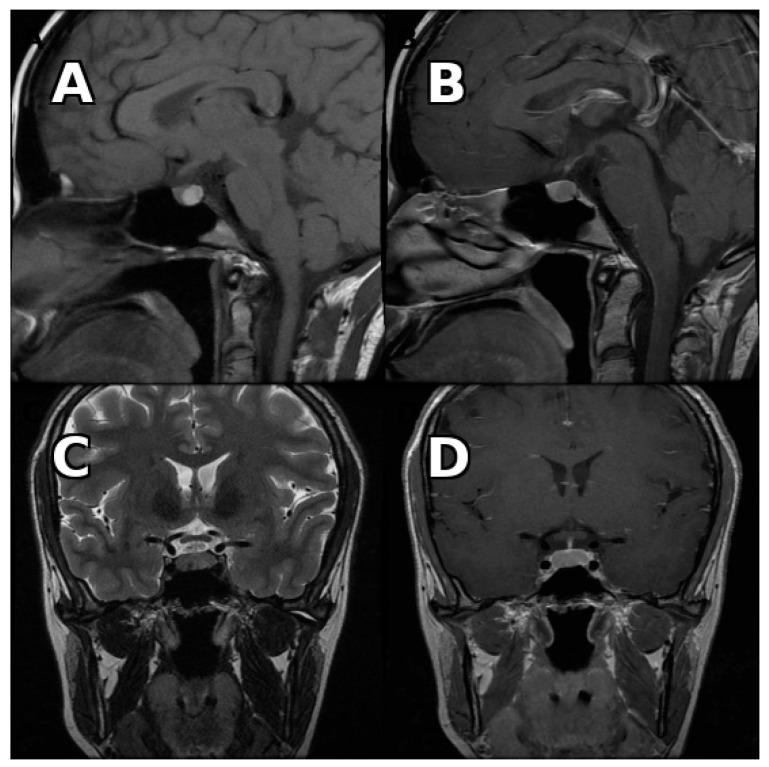
Pituitary MRI of a patient with Rathke’s cleft cyst. (**A**) Sagittal T1-weighted image demonstrating spontaneous hyperintensity of the cystic lesion. (**B**) Contrast-enhanced sagittal T1-weighted image demonstrating no enhancement. (**C**) Coronal T2-weighted image demonstrating midline localization. (**D**) Contrast-enhanced coronal T1-weighted image demonstrating the midline position without enhancement.

Similarly, spontaneous hyperintensity on T1-weighted images and midline location on sagittal and coronal images were significantly associated with Rathke’s cleft cyst (*p* = 0.016, <0.001, and <0.001, respectively) (Figure 5).

In contrast, signal change and size change during follow-up imaging did not differ significantly between the two groups (*p* = 0.126 and *p* = 0.23, respectively) (Table 4).

### 3.3. Maximum Lesion Diameter

The maximum lesion diameter was 14.07 ± 4.87 mm (range: 10–32 mm) in the cystic pituitary adenoma group and 12.07 ± 1.62 mm (range: 10–15 mm) in the Rathke’s cleft cyst group. The difference between groups did not reach statistical significance (*p* = 0.138) (Table 5). The values presented in Table 5 are consistent with the descriptive statistics reported in the text.

**Figure 6:**
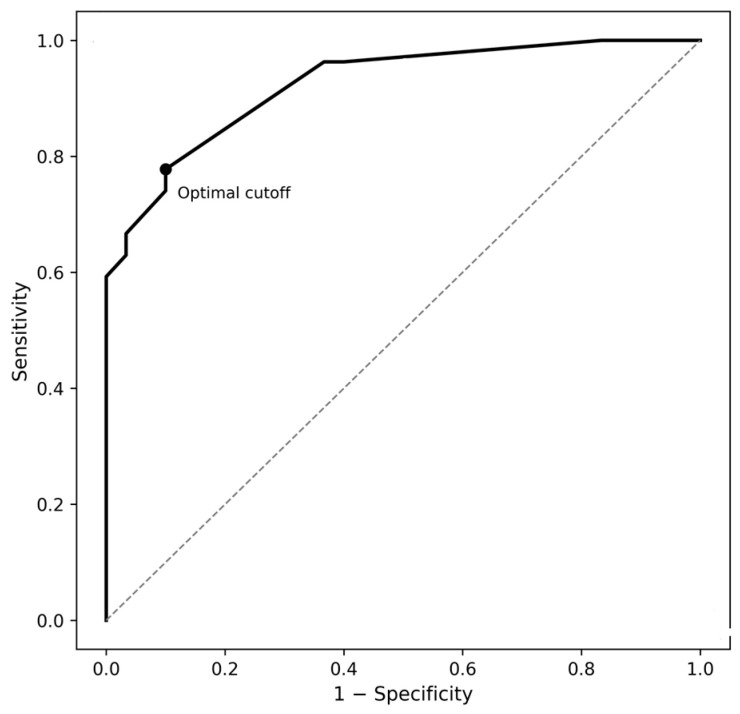
Receiver operating characteristic (ROC) curve for maximum lesion diameter in differentiating cystic pituitary adenoma from Rathke’s cleft cyst, demonstrating limited discriminative performance (area under the curve (AUC) = 0.614). The dashed line represents the line of no discrimination (AUC = 0.5).

Receiver operating characteristic (ROC) curve analysis demonstrated limited discriminative ability of maximum lesion diameter for differentiating cystic pituitary adenoma from Rathke’s cleft cyst (AUC = 0.614; 95% confidence interval: 0.464–0.764; *p* = 0.144) (Figure 6).

### 3.4. Multivariable Logistic Regression and Model Performance

Multivariable logistic regression analysis identified septation and fluid–fluid level as independent predictors of cystic pituitary adenoma (odds ratio (OR): 48.48; 95% confidence interval (CI): 3.91–601.49; *p* = 0.002 and OR: 48.87; 95% CI: 2.29–1042.96; *p* = 0.013, respectively). In contrast, spontaneous T1 hyperintensity was independently associated with Rathke’s cleft cyst (OR: 0.05; 95% CI: 0.005–0.49; *p* = 0.010). T2 hypointense rim (OR: 4.25; 95% CI: 0.40–45.37; *p* = 0.231) and intracystic nodule (OR: 0.50; 95% CI: 0.03–8.40; *p* = 0.634) were not statistically significant in the multivariable model (Table 6).

The final diagnostic prediction model demonstrated excellent discrimination, with an area under the curve (AUC) of 0.91. Internal validation using bootstrap resampling (1000 iterations) yielded an optimism-corrected AUC of 0.88, indicating stable model performance with minimal evidence of overfitting. The small reduction between the apparent AUC and the optimism-corrected AUC suggests that model performance remained relatively stable across bootstrap resampling iterations.

The Hosmer–Lemeshow goodness-of-fit test demonstrated good calibration, and calibration plots showed close agreement between predicted and observed probabilities. Decision curve analysis demonstrated a positive net clinical benefit across threshold probabilities ranging from 10% to 70%, supporting the potential clinical applicability of the model.

The optimal probability cutoff determined by the Youden index was 0.49, yielding a sensitivity of 77.8% and a specificity of 90.0%.

### 3.5. Prediction Model Equation

The final logistic regression model incorporated fluid–fluid level, hypointense rim on T2-weighted images, septation, intracystic nodule, and spontaneous T1 hyperintensity. The model can be expressed in logit form as follows:

logit(P) = −0.33 + 3.88(septation) + 3.89(fluid–fluid level) + 1.45(T2 hypointense rim) − 0.69(intracystic nodule) − 2.91(spontaneous T1 hyperintensity),

where β_0_ denotes the intercept of the model.

The predicted probability of cystic pituitary adenoma can be calculated using the following equation:

P = 1/(1 + e^(−logit(P))).

**Figure 7:**
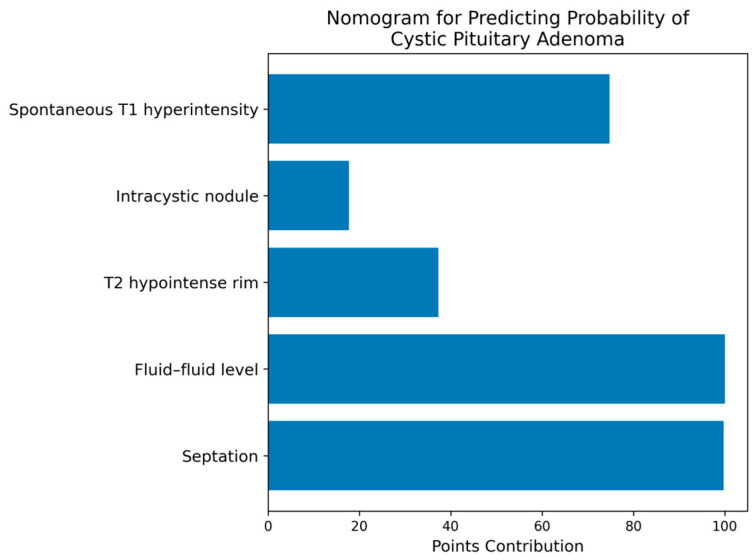
Nomogram for estimating the probability of cystic pituitary adenoma based on MRI predictors included in the final multivariable logistic regression model, providing an intuitive graphical tool for estimating individual patient probability using routinely available MRI features.

To facilitate clinical application of the prediction model, a nomogram based on the final logistic regression model was constructed to estimate the probability of cystic pituitary adenoma using the identified MRI predictors (Figure 7).

## 4. Discussion

In this study, we developed and internally validated an MRI-based diagnostic prediction model to differentiate cystic pituitary adenoma (CPA) from Rathke’s cleft cyst (RCC). The final multivariable model demonstrated excellent discriminative performance (AUC 0.91), with stable optimism-corrected performance after bootstrap validation (AUC 0.88), indicating minimal overfitting and good internal validity.

Consistent with previous literature, fluid–fluid level, hypointense rim on T2-weighted images, and septation were independently associated with CPA. Park et al. [11], in a pathology-correlated study, similarly reported that fluid–fluid level, septation, and off-midline location were significantly more common in cystic adenomas. Tavakol et al. [10], in a large retrospective cohort, also demonstrated a strong association between fluid–fluid levels and adenomatous lesions. The present findings reinforce these established imaging markers by demonstrating their independent contribution within a multivariable predictive framework rather than as isolated features. These results are also consistent with findings reported in larger neuroradiological cohorts, in which fluid–fluid levels and internal septations were identified as among the most reliable MRI features favoring cystic pituitary adenomas over Rathke’s cleft cysts. Such studies have emphasized that these imaging characteristics remain important diagnostic indicators, even when evaluated in heterogeneous patient populations.

In contrast, intracystic nodules and spontaneous T1 hyperintensity were independently associated with RCC. Intracystic nodules have been widely described as characteristic findings of RCC, with reported frequencies ranging from 17% to 78% in different cohorts [14,15,16]. Studies by Byun et al. [12], Wen et al. [15], and Binning et al. [14] emphasized the diagnostic importance of these nodules, particularly when demonstrating typical T1 hyperintensity and T2 hypointensity. The present results confirm their diagnostic relevance and demonstrate their independent contribution within a combined predictive model. The prevalence of intracystic nodules observed in the present study falls within the range reported in larger imaging cohorts, although some variability across studies may reflect differences in patient selection, imaging protocols, and diagnostic criteria used for nodule identification.

Spontaneous T1 hyperintensity, likely reflecting protein-rich cyst content, was also significantly associated with RCC. Although prior studies have described variable T1 signal intensity in RCC [4], relatively few investigations have evaluated its independent diagnostic value in comparison with CPA. These findings suggest that this feature may provide incremental diagnostic value when interpreted alongside other MRI characteristics.

Lesion location analysis further supported previous observations. RCCs were significantly more likely to demonstrate midline localization on sagittal images, whereas CPAs more frequently exhibited paramedian positioning. Although midline localization has traditionally been described as typical for RCC [4], our study quantitatively confirms its relevance within a predictive modeling framework.

Maximum lesion diameter did not demonstrate meaningful discriminative ability (AUC 0.614), indicating that lesion size alone is insufficient for reliable differentiation. This finding is consistent with previous studies reporting substantial size overlap between CPA and RCC [13]. Furthermore, comparison of maximum lesion diameters between the two groups showed no statistically significant difference, reinforcing the limited diagnostic value of lesion size as an isolated parameter. The values presented in Table 5 are consistent with those reported in the Section 3, confirming the internal consistency of the dataset. Similarly, previous studies including larger patient cohorts have reported considerable overlap in lesion size between cystic pituitary adenomas and Rathke’s cleft cysts, suggesting that lesion diameter alone has limited value for reliable differentiation of these entities.

Unlike earlier descriptive imaging studies that evaluated MRI findings individually, the present study proposes and internally validates a multivariable diagnostic prediction model that integrates several imaging features simultaneously. By combining septation, fluid–fluid level, T2 hypointense rim, intracystic nodule, and spontaneous T1 hyperintensity within a single predictive framework, the model enables a more objective estimation of diagnostic probability rather than reliance on isolated imaging signs. This integrated approach may facilitate more standardized preoperative evaluation and support clinical decision-making in neuroradiological practice.

In the final multivariable model, T2 hypointense rim and intracystic nodule did not reach statistical significance. However, these variables were retained because of their well-established diagnostic relevance in prior neuroradiological studies and their potential contribution to the clinical interpretability of the prediction model. Retaining these predictors allowed the model to incorporate imaging features that may still provide complementary diagnostic information when considered in combination with other variables.

Furthermore, the model incorporates multiple aspects of predictive performance, including discrimination, calibration, and decision curve analysis. The results demonstrated good calibration and a positive net clinical benefit across clinically relevant threshold probabilities, suggesting potential clinical applicability in preoperative decision-making. Because the model relies exclusively on conventional MRI sequences, it may be readily implemented in routine neuroradiological practice without specialized post-processing techniques. To further enhance clinical applicability, the prediction model was translated into a nomogram that enables clinicians to visually estimate the probability of cystic pituitary adenoma based on the MRI predictors included in the model. Such graphical tools may facilitate the practical use of prediction models in routine neuroradiological decision-making.

Although the apparent AUC of 0.91 suggests excellent model discrimination, such high performance in relatively small datasets may raise concerns regarding potential overfitting. In the present study, bootstrap-based internal validation demonstrated only a modest reduction in performance after optimism correction (AUC 0.91 vs. optimism-corrected AUC 0.88). This limited decline in model performance suggests that the prediction model remained relatively stable across bootstrap resampling iterations and supports the robustness of the estimated predictive performance.

The relatively wide confidence intervals observed for certain predictors likely reflect the modest sample size and limited event counts for some imaging features. To mitigate potential model instability, the number of predictors included in the final multivariable model was deliberately restricted according to the events-per-variable principle. This strategy was applied to maintain model stability and reduce the likelihood of regression coefficient overfitting. Internal validation using bootstrap resampling further demonstrated consistent model performance, supporting the overall robustness of the predictive estimates.

Nevertheless, the relatively wide confidence intervals indicate that the precision of some effect estimates remains limited, and these associations should therefore be interpreted with appropriate caution. The observed variability likely reflects statistical uncertainty related to the modest sample size rather than true instability of the predictive model. Similar sample sizes have been reported in several previous neuroradiological studies evaluating MRI characteristics of cystic sellar lesions.

External multicenter validation is warranted before routine clinical implementation. These findings are clinically relevant given that differentiating cystic pituitary adenoma from Rathke’s cleft cyst on MRI remains challenging because of overlapping imaging characteristics [16].

In addition, because histopathological confirmation was not available for all patients and some cases were classified based on clinical and imaging follow-up combined with a previously published diagnostic algorithm, a degree of diagnostic misclassification and potential classification bias cannot be completely excluded. A sensitivity analysis restricted to histopathologically confirmed cases could not be performed due to the limited sample size. This methodological limitation should therefore be considered when interpreting the findings of the present study.

Taken together, the present findings suggest that integrating multiple MRI features within a structured predictive framework may improve the preoperative differentiation between cystic pituitary adenoma and Rathke’s cleft cyst. However, these results should be interpreted cautiously until further validation studies with larger cohorts become available.

Importantly, all predictors included in the final model are routinely available on conventional pituitary MRI sequences, supporting the feasibility of applying this approach in routine neuroradiological practice without the need for additional imaging techniques.

Future multicenter studies with larger cohorts and external validation are needed to confirm the generalizability of these findings and to further refine MRI-based diagnostic strategies for cystic sellar lesions.

## 5. Limitations

Several limitations should be acknowledged. First, the retrospective single-center design may introduce selection bias. In addition, because cases were identified retrospectively through the Picture Archiving and Communication System (PACS), a degree of selection bias related to database-based case identification cannot be entirely excluded.

Second, histopathological confirmation was unavailable for all cases. However, inclusion of patients with adequate clinical and radiological follow-up was intended to minimize potential diagnostic misclassification. For patients without histopathological confirmation, lesion classification was based on clinical findings, endocrine laboratory results, and longitudinal imaging follow-up. The median follow-up duration was 12 months (range: 6–24 months), during which imaging stability or characteristic lesion evolution was considered supportive of the final diagnosis. Nevertheless, the use of imaging follow-up combined with an imaging-based diagnostic algorithm may introduce a potential risk of diagnostic misclassification and partial circular reasoning, as some imaging characteristics evaluated in the analysis may also contribute to the clinical consensus classification. This limitation should therefore be considered when interpreting the findings.

Furthermore, the inclusion criteria focusing on cystic sellar lesions without contrast-enhancing solid components may have introduced a degree of spectrum bias, as the imaging characteristics of the study population may not fully represent the entire spectrum of cystic pituitary lesions encountered in broader clinical practice.

Third, although internal validation was performed using bootstrap resampling to reduce model optimism, external validation in independent multicenter cohorts is necessary before broader clinical adoption.

Finally, the relatively modest sample size may limit the detection of smaller effect sizes and may also influence the stability of multivariable estimates. However, the number of predictors included in the final model was deliberately restricted in order to reduce the risk of model overfitting and to maintain an acceptable events-per-variable ratio.

## 6. Conclusions

In conclusion, an MRI-based multivariable diagnostic prediction model incorporating septation, fluid–fluid level, a T2 hypointense rim, intracystic nodule, and lesion localization demonstrated high discriminative performance for differentiating cystic pituitary adenoma from Rathke’s cleft cyst. The model showed good calibration and internal validity and may assist clinicians in the preoperative evaluation of cystic sellar lesions.

Accurate differentiation between cystic pituitary adenoma and Rathke’s cleft cyst is essential for appropriate clinical management. Because the predictors included in the model are derived from routinely available conventional pituitary MRI sequences, the proposed approach may be readily applied in routine neuroradiological practice and may help reduce unnecessary surgical intervention in patients with Rathke’s cleft cyst. However, external validation in larger multicenter cohorts is required before routine clinical implementation.

## Figures and Tables

**Table 1:** Demographic and clinical characteristics of the study population.

Variable	Cystic Pituitary Adenoma (*n* = 27)	Rathke’s Cleft Cyst (*n* = 29)	*p*-Value
Sex, *n* (Male/Female)	8/19	5/24	0.273
Age, years (mean ± SD)	39.7 ± 15.1	32.8 ± 9.9	0.144

Note: Continuous variables are presented as mean ± standard deviation (SD).

**Table 2:** Interobserver agreement for evaluated MRI features.

MRI Feature	Kappa (κ)	Agreement
Fluid–fluid level	0.923	Excellent
T2 hypointense rim	1.000	Excellent
Septation	0.879	Excellent
Intracystic nodule	0.926	Excellent

Note: Interpretation based on Landis and Koch criteria for kappa values.

**Table 3:** Comparison of MRI features between cystic pituitary adenoma and Rathke’s cleft cyst (Part 1).

Feature	CPA n (%)	RCC n (%)	*p*-Value
Fluid–fluid level	7 (25.9)	1 (3.4)	0.023
T2 hypointense rim	9 (33.3)	2 (6.9)	0.018
Septation	17 (63.0)	1 (3.4)	<0.001
Intracystic nodule	4 (14.8)	19 (65.5)	<0.001

Note: Data are presented as number of patients with percentages in parentheses. Abbreviations: CPA, cystic pituitary adenoma; RCC, Rathke’s cleft cyst.

**Table 4:** Comparison of MRI features between cystic pituitary adenoma and Rathke’s cleft cyst (Part 2).

Feature	CPA n (%)	RCC n (%)	*p*-Value
Spontaneous T1 hyperintensity	12 (44.4)	22 (75.9)	0.016
Midline sagittal location	6 (22.2)	23 (79.3)	<0.001
Paramedian coronal location	24 (88.9)	4 (13.8)	<0.001

Note: Data are presented as number of patients with percentages in parentheses. Abbreviations: CPA, cystic pituitary adenoma; RCC, Rathke’s cleft cyst.

**Table 5:** Maximum lesion diameter in patients with cystic pituitary adenoma (CPA) and Rathke’s cleft cyst (RCC).

Group	Maximum Lesion Diameter (mm)	*p*-Value
CPA	14.1 ± 4.9	0.138
RCC	12.1 ± 1.6	0.138

Note: Data are presented as mean ± standard deviation (SD).

**Table 6:** Multivariable logistic regression analysis for differentiating cystic pituitary adenoma from Rathke’s cleft cyst.

Variable	OR	95% CI	*p*-Value
Septation	48.48	3.91–601.49	0.002
Fluid–fluid level	48.87	2.29–1042.96	0.013
T2 hypointense rim	4.25	0.40–45.37	0.231
Intracystic nodule	0.50	0.03–8.40	0.634
Spontaneous T1 hyperintensity	0.05	0.005–0.49	0.010

Abbreviations: OR, odds ratio; CI, confidence interval.

## Data Availability

Data are contained within the article.

## Abbreviations

The following abbreviations are used in this manuscript:

AUC	Area Under the Curve
CI	Confidence Interval
CPA	Cystic Pituitary Adenoma
FOV	Field of View
FSE	Fast Spin Echo
MRI	Magnetic Resonance Imaging
OR	Odds Ratio
PACS	Picture Archiving and Communication System
RCC	Rathke’s Cleft Cyst
ROC	Receiver Operating Characteristic
SI	Signal Intensity
TE	Echo Time
TR	Repetition Time
